# How to Grow Beautiful

**Published:** 1888-08-25

**Authors:** 


### How to Grow Beautiful.

It has been said that no woman knows that she is plain. She knows that the girl next door is ugly, or the lady over the way a perfect fright, but she never does more than suspect her own lack of beauty. Nevertheless in many female breasts the suspicion is so very strong that there is no quicker way for an enterprising chemist to make a fortune than the invention of a new cosmetic. Perhaps we ought to say a new name, for practically these wonderful mixtures are very much alike, and the novelty lies in the appellation. This year's *Poudre d'Aphrodité* is very much like last year's "Bloom of Hebe," and is about equally successful in restoring the lustre of youth to cheeks whose roses time has dimmed, and brows whose smooth whiteness care has furrowed. That is, they each cover up natural defects, and make the women who use them persistently, become a species of whited sepulchre—a ghastly simulacrum of youth, that may "pass in a crowd," but is not adapted to the close and often unflattering scrutiny of the home circle. But as a rule these embellishments are used in the secret of the dressing-room, and the one thing in which the advertisements of all the nostrums agree is that they will be sent "post free, and secure from observation," for about ten times the price of the ingredients.

That has been the rule hitherto, but *nous avons changé tout cela*. Woman, lovely or otherwise, has lost the habit of telling her wishes in shy whispers; she announces them from the platform, and placards them in the newspapers; and she has now declared her intention of seeking beauty as openly as she does Parliamentary representation. As is common nowadays, the sun of progress rises in the west, and it is in America that a physician has just set up as avowedly a "beauty doctor." The new practitioner is a lady. This is fitting. The best of men—poor things!—can't see the real seriousness of red hair, a dull complexion, or a figure over plump or over thin. They think that if her lungs and heart are free from disease, and her liver is in as good condition as a long course of corset-wearing will permit, the patient is in perfect health, and tell her "not to worry." Perfect health—with freckles on her face, or a wart on her hand! The lady doctor, if she is at all sympathetic, will not lack patients.

According to the published accounts she is a very stern doctor, however, and throws her patients up if they will not obey her prescriptions, which are generally extremely sensible. In fact, she tells the laws of health to women who have been guilty of every fashionable folly that can injure the constitution, and insists on their obeying them, knowing that they will do so to gain beauty when they would not to acquire health. So long as the "beauty-doctor" confines her prescriptions to frequent baths, simple food, and regular habits of life, she will do nothing but good, and we wish her all success.

There is a part of beauty, however, with which even the American doctor does not deal, the only method, we may inform her and her patients, of preserving that charm which is worth more than regularity of feature or freshness of complexion. The latter will go in spite of all the beauty prescriptions in the world, and crows'-feet and wrinkles will spoil the most exquisite lines of feature. And yet there should be no ugly age. We cannot agree with Theophile Santile, that at the age of thirty all women should commit suicide in despair at losing their looks: the beauty that will not last a woman through half her life is but a poor thing. We do not wish to usurp the preacher's place, but we devoutly believe in Kingsley's theory that the soul dominates the body, and with advancing years shines more and more clearly through it. There never yet was a beautiful soul lodged permanently in a displeasing frame; and there is many a woman who in her youth attained only the "bloom of ugliness" who finds in middle life and old age that she wins more love than her once fairer sisters.

